# System Level spatial-frequency EEG changes coincident with a 90-day cognitive-behavioral therapy program for couples in relationship distress

**DOI:** 10.1007/s11682-014-9319-7

**Published:** 2014-10-02

**Authors:** Donald R. DuRousseau, Theresa A. Beeton

**Affiliations:** 1 PEAK Neurotraining Solutions Inc., Herndon, VA USA; 2 Loudoun Family and Relationship Counseling, Inc., Leesburg, VA USA

**Keywords:** Quantitative EEG, Imago relationship therapy, Cognitive behavioral interventions, Resting state networks, Spatial-frequency EEG

## Abstract

Evaluating relationship intervention programs traditionally involves the use of self-report surveys or observational studies to assess changes in behavior. Instead, to investigate intervention-related changes in behavior, our study evaluates spatial-frequency electroencephalography (EEG) patterns from the brains of couples participating in an Imago Relationship workshop and 12 weeks of group counseling sessions lasting approximately 90 days. This explorative study recorded 32-channel EEGs from nine committed distressed couples prior to, during and immediately following the Imago Relationship Therapy program. A repeated measures *t*-Test approach was applied to investigate if significant group level brain pattern changes could be identified in key resting state networks in the brains of the participants that could be correlated with changes in relationship outcome. The study results show that significant reductions in EEG power in the alpha_2_, beta_3_ and gamma bands were evident in the averaged brain activity in the pre-frontal, frontal and temporal-parietal cortices that are anatomically associated with the frontal executive, default mode and salience networks of the human brain. Our current understanding of system level neural connectivity and network dynamics strongly indicates that each of these systems is integrally required in learning and implementing a complex communication process taught in the Imago intervention. Thus, a high degree of hemispheric lateralization is consistent with our understanding of language function and mood regulation in the brain and is consistent with recent research into the use of resting frontal EEG asymmetry as an indicator of behavioral changes in distressed couples undergoing a program for relationship improvement. Although preliminary, these results further indicate that the EEG is an inexpensive and easily quantifiable measure, and possibly predictor, of behavioral changes in response to a cognitive behavioral intervention.

System level spatial-frequency EEG changes coincident with a 90-day cognitive-behavioral therapy program for couples in distress.

Relationship distress has a strong connection to an individual’s level of mental and physical health problems (Whisman and Uebelacker 2003, 2006). Programs that help couples in distress are valuable because people are more likely to have physiological and psychological health problems if they are in relationship distress. Well over 70 % of psychotherapists report that they provide couples counseling indicating a strong need for this type of intervention (Orlinsky and Ronnestad 2005). Research indicates that therapy for couples is a viable and useful endeavor for couples seeking relationship improvement (Lebow et al. 2012). One recent study evaluated fMRI patterns of unhappily married people and explored how they experienced pain before and after couple’s treatment. The participants experienced less pain with a better sense of connection to their partner (Johnson et al. 2013). While this study reports on brain region activation, it does not reveal the action of brain activity changes that emerged from the treatment program. Most current research related to couple’s therapy programs do not evaluate the specific impact on functional brain activity patterns of the couples before, during, and after the course of treatment. Thus, this study provides an exploratory investigation of the system level spatial-frequency changes over the course of the cognitive behavioral treatment (CBT) program known as Imago Relationship Therapy (IRT).

## Background

Investigating EEG pattern changes is a sound endeavor because it provides anatomically based insights about the true impact of relationship interventions at the network level of the brain and may provide keys as to how these changes correlate to symptom relief and the achievement of improved relationship satisfaction. Functional anatomical and metabolic neuroimaging research has provided considerable detail in terms of identifying executive, attentional and emotional processing networks in the brain. Regions involved in emotional regulation include the amygdala, hippocampus, cingulate and medial frontal cortices; all part of limbic system (Calu et al. 2010; Hermans et al. 2014). Additionally, conceptual executive planning and decision making occur in the orbital-frontal, fronto polar, insular, cingulate and medial frontal cortices (Boorman et al. 2009; Kluger 2009). Consistently, fMRI and EEG neuroimaging research provides excellent detail concerning hubs and spokes connectivity with models of seizure disorders (Prinz 2008; Vogels and Abbott 2009). More recent observations reveal and indicate broad network reorganization in key neural systems during task related and resting state activities (Yan et al. 2009; Dipoppa and Gutkin 2013).

Further, Buzsáki (2006) has offered keen insights into how communication and cooperation are achieved in the brain at the cellular and small network levels using the mechanism of cross-frequency coupling of the EEG waveforms. Such evidence provides support to the concept that specific brain centers and interconnected neural networks are involved when learning to use an empathic dialogue process and reorganization of activity among these distributed brain hubs is a necessary prerequisite for cognitive and behavioral changes to occur in the couple’s relationship system dynamic (Smith et al. 2009; Acevedo et al. 2012). Thus these works and even more recent research provide convincing evidence that the brain is a distributed and interconnected system of systems, and as such, if changes in cognitive strategy are to occur, the central executive (CE) and Salience network (SN) must be involved (Jung et al. 2013; Doll et al. 2013). In this study, we rely upon this concept to support our investigation of how large scale brain Resting State Networks (RSN) might be reorganized when subjects learn to change their communication process and practice the use of Imago dialogue to develop increased satisfaction with their partner.

Brain systems primarily involved in learning and memory processes are located in the frontal and temporal lobes (Delgado and Dickerson 2012) while the emotional interaction with memory involves the midline cingulate gyrus, as well as the amygdala and hippocampus located in each temporal lobe (Liao et al. 2010). Cognitive behavioral therapy (CBT) interventions have been used to treat anxiety and stress disorders and EEG results for these studies show changes in cross frequency coupling, showing greater power in right over left resting frontal brain activity from pre- to post-treatment (Moscovitch et al. 2010; Miskovic et al. 2011).

Our results suggest the involvement of several brain systems involved in the regulation of executive, mood, and decision making functions, where the locations of greatest peak difference in EEG band power were consistent across subjects at a few narrow frequencies when comparing the before vs. after intervention datasets. Our approach assumed that coordinated brain activity involved in organizing higher order processes are carried out through the process of cross frequency coupling operating at a network systems level. Therefore, in our investigation we anticipated significant frequency band changes in a small number of key interconnected brain regions co-involved in language, dialogue, executive control and empathy (Lau et al. 2013; Morgan and Soltesz 2008; Watt 2007; Cox et al. 2012). Bolstered by an ever-growing wealth of neuroscience literature from both the molecular imaging and CBT fields in support of our approach, we examined spatial-frequency EEG changes from nine married couples before, during, and after participation in the Imago workshop and 90-day group therapy program.

## Method

### Participants

Ten couples were recruited for this study and signed informed consent forms prior to joining the investigation. One couple dropped out after attending the preliminary workshop for couples. All of the participants were assessed using the Dyadic Adjustment Scale (DAS) to understand if the couples were in a distressed relationship. Two of the couples scored as adjusted (scores above 96) and the remaining seven couples scored in the distressed range below 96. The participants were all self-identified as Caucasian, with the exception of one participant identifying as Hispanic/Asian. The youngest participant was 26 and the oldest participant was 65. The average age of participants was 47. The income levels of the participants averaged over $100,000. The lowest income level was $10,000 and the highest was over $350,000. All of the couples were in committed relationships, with one couple indicating that they were currently separated. Couples had been in-relationship for an average of 17 years, with the shortest length of relationship being 3 years and the longest 42 years. Of the eighteen people participating in the study, nine claimed no spiritual affiliation, one was Jewish, four were Sikhs, and four were Christian. Exclusionary criteria required couples to be in no other treatment program outside of the counseling intervention. None of the participants reported using any psychotropic medication or prohibited drugs. All of the participants were given written instructions and received all study information in accordance with standard health and human subject research protocols.

### Clinical treatment program

Participants completed a 20 h standardized Imago Relationship workshop, *Getting the Love You Want* (GTLYW), developed by Harville Hendrix and Helen La Kelly Hunt (1988). In addition to attending the workshop, the individuals all participated in 12 sessions of group-based couples counseling. The sessions were designed to help the couples practice listening and dialog skills and processes learned in the workshop. Imago has been described as a CBT program that invokes emotional and empathic connections in marital partners (Berger and Hannah 1999) For more information about the GTLYW program see www.gettingtheloveyouwant.com.

### Self-report measures

Relationship stress was measured through the use of the DAS (Spanier 1976). Participants completed this survey at the same time their EEG was recorded; prior to taking the workshop, after 6 weeks of group counseling and then immediately after completing 12 weeks of group counseling.

### EEG recording and data reduction

For each subject, 32 single-ended EEG sensors plus linked ear references were located on the head according to the International 10–20 placement system using a stretchable elastic cap made by Compumedics Inc. In addition to the EEG sensors, 2 bipolar sensor pairs were located at the left and right outer canthus for the horizontal electrooculogram (HEOG) and above and below the left eye to measure vertical eye movement and blinks (VEOG). Another bipolar sensor pair was also located at the back of the neck near the base of the skull to measure both the electrocardiogram (ECG) and neck muscle activity (EMG) for a total of thirty-six (36) physiological channels. The added bipolar signals were used to monitor the amount of contamination present in the EEG during recordings, and to ensure that these artifacts were not included in the EEG data that were manually selected for analysis purposes. For all subjects, the EEG was recorded under two at-rest conditions: a 20-min eyes closed (EC) recording and a 10-min eyes open (EO) recording. For each condition, a series of three repeated-test recordings were made from the eighteen subjects at the following intervals; prior to (Baseline (BL), midway through (MID), and immediately following (POST) the 12-week Imago CBT program.

**Fig. 1:**
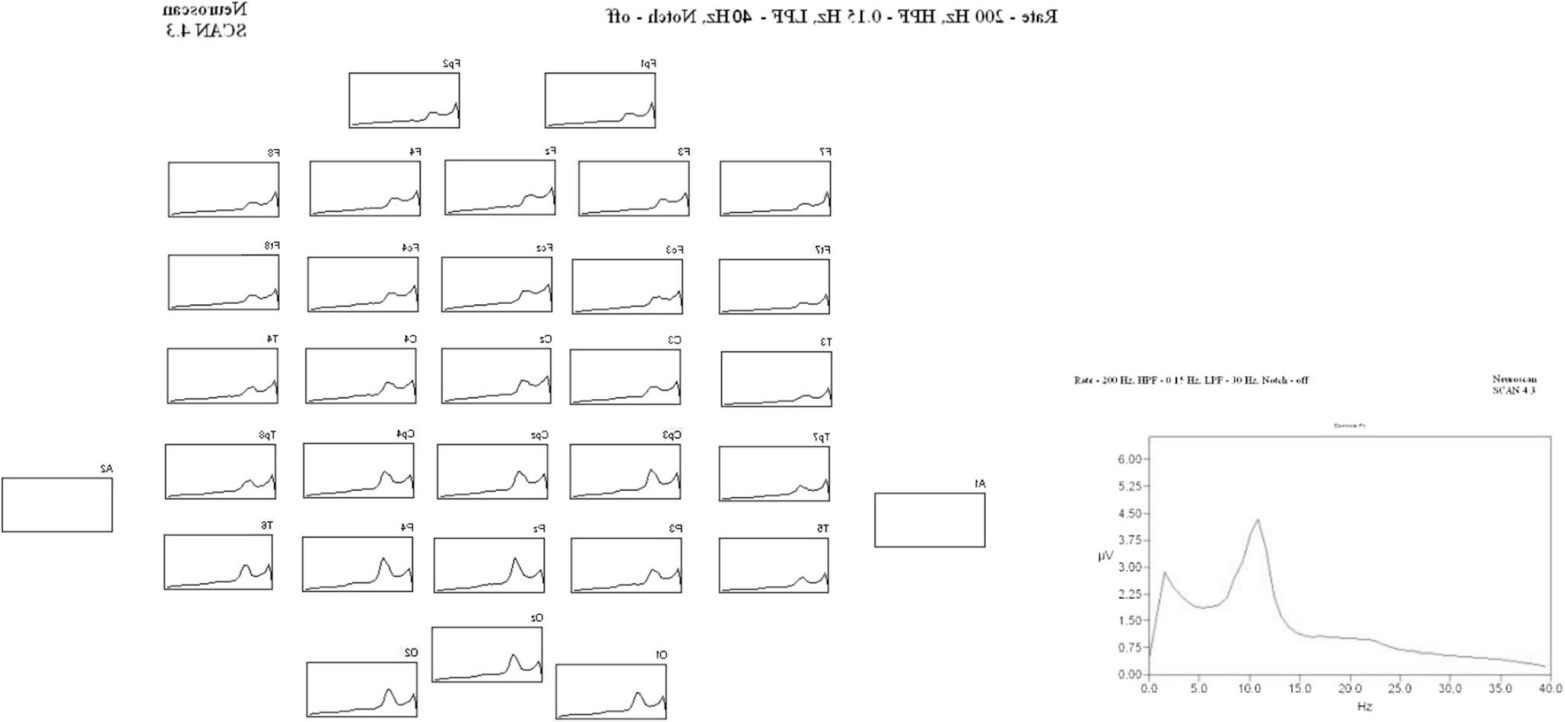
Individual averaged data set. Individual averaged dataset made from 1.3-s epochs of uncontaminated eyes-closed resting EEG data from 32 electrodes. The vertical axis displays EEG power in uV^2^ and the horizontal axis indicates the frequency of the EEG from 0 to 40 Hz. The insert shows the spectral power graph for the Pz sensor

**Fig. 2:**
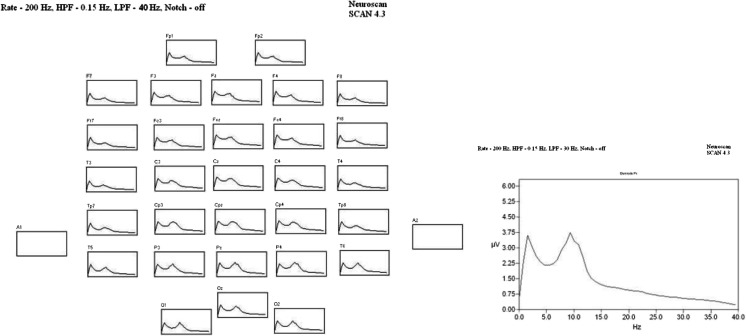
Group averaged EC data set. Group averaged dataset (*N* = 18) indicating spatial-frequency activity from 1.3-s epochs of uncontaminated EC resting EEG data from 32 electrodes. The vertical axis displays spectral power in uV^2^ and the horizontal axis indicates the frequency of the EEG from 0 to 40 Hz. The insert shows the group peak-to-peak power graph for the Pz sensor

The referential and bipolar data were amplified and recorded using an FDA-cleared clinical EEG system made by Embla Medical Systems Inc., Broomfield, CO. All data were sampled at 200 Hz with a high-pass filter of 0.15 Hz and low-pass filter of 70 Hz. Visual inspection and a manual editing tool were used to select only uncontaminated data that had no eye, heart, muscle, movement, or environmental noise. The selected clean data samples were further processed using zero-phase band-pass filters set from 1 Hz (48 dB/octave) to 40 Hz (96 dB/octave). Next, for each subject and condition, the filtered datasets of varying lengths were segmented into contiguous 1.3 s long epochs, where the number of artifact free samples ranged from 381 to 1,074 for the 20-min EC conditions (BL, MID, and POST) and from 100 to 455 for the 10-min long EO conditions. The epoched files were then used to create amplitude scaled frequency domain averages for each subject’s EC and EO datasets using a Fast Fourier Transform (FFT) with a Hamming window length of 10 %, providing better than 1 Hz. resolution (0.8 Hz). A resulting individual dataset is shown in Fig. 1 for each of the 32 EEG channels from a single subject. The plots in the figure show the average peak-to-peak power from the 20-min recording in microvolts squared (uV^2^) on the vertical axis at each frequency from 0 to 40 Hz (horizontal axis). Finally, the individual averages were combined within sessions and conditions to create six (6) Group Averaged EEG files used for difference testing and statistical evaluation. Figure 2 shows a Group Averaged EC dataset made from all 18 subjects from the third test recording (EC_POST).

For each subject, the three EC and EO averages (periods t^1^, t^2^ and t^3^) were made from the BL, MID, and POST recording sessions (3 test sessions × 2 conditions = 6 averages per subject). The average number of 1.3 s epochs used for the analysis of the EC condition data was 720 (equivalent to 15.6 min of EEG data) with a range of 693, std. dev. = 143, and std. error = 19.5. For the EO sessions, the average of 1.3 s samples was 290 (6.2 min of EEG data), with a range of 355, std. dev. = 87, std. error = 12.

To determine if a significant difference existed in the spatial-frequency EEG attributable to the CBT intervention, we examined the test-retest reliability of the averaged brain activity differences during the EC and EO conditions over the 3 test sessions using a paired *t*-Test methodology. To accomplish this, each subject’s EO and EC Average Files, made from several hundred samples of noise-free data, were used to create six Difference Files representing changes from the first-half (∆^1^), second-half (∆^2^) and overall study periods (∆^3^). The six Difference Files (3 EO and 3 EC) were made for each subject by subtracting: 1) the mid-phase data from the pre-intervention baseline (∆^1^ = BL-MID) to evaluate the first-half differences, 2) the post-intervention data from the mid-intervention (∆^2^ = MID-POST) to detect any second-half differences and 3) the post from the baseline condition (∆^3^ = BL-POST) to measure the overall brain activity changes.

## Results

### Dyadic adjustment scale

Individual DAS scores were averaged as couple scores. Table 1 presents the individual couple scores for study participants. Two of the couples started the workshop with DAS scores indicating satisfactory adjustment. Seven of the couples were in a distressed situation. At the mid-point of the study, four of the couples were distressed and five reported as adjustment. The same scores held for the post reporting phase of the study. The two couples who indicated that they were adjusted stayed adjusted showing a five-point increase. Two of the couples in distress stayed in distress and even lost significant points in adjustment. One of the distressed couples stayed distressed but gained 16 points. The other three couples that started out in distress but improved showed increases up to 32 points. One couple in distress showed the same score from start to finish. The scores ranged from losing 22 points in adjustment to gaining 32 points in dyadic adjustment.

**Table 1:** DAS scores for couples pre-, mid-, and post-Imago intervention

Couple	BL	MID	POST
A	69	87.5	85.5
B	109	99	114
C	93	84	71.5
D	91.5	90	77
E	110	110.5	115
F	96	99.5	110.5
G	94.5	118	127
H	95.5	97	100
I	94	96	94.5

### Spatial-frequency EEG difference results

Three dimensional mapping of the temporal and frequency components of the EEG indicated a clear differential pattern of activity in the CE, DM, and SN networks during and after the CBT intervention with significant involvement of the right fronto-polar, ventral-lateral and dorsal-medial aspects of the PFC (vlPFC and dmPFC) in the alpha_2_ band (9–13 Hz), as well as in the central and right superior-parietal and temporal-parietal cortices in the beta_3_ frequencies (14–25 Hz). The right PFC, parietal, and temporal foci remained fixed into the gamma band out to 40 Hz. The course of these difference patterns progressed over the intervention by showing no real changes during the first-half of the program to demonstrating significant differences during the second half of the intervention, as well as overall. Most importantly, the significant spatial-frequency activations were consistent with our understanding of the cortical locations and networks involved in dialog, executive control, decision-making, and emotional regulation; all prerequisites of the CBT intervention.

**Fig. 3:**
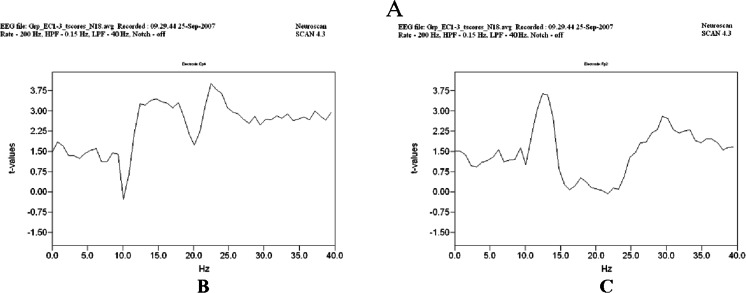
Group averaged t-value datasets (**a**) Group averaged t-Value dataset (*N* = 18) testing the significance of the EC spatial-frequency pattern changes over the entire program from Baseline to Post (∆^3^) using a paired *t*-Test of the means (df = 17, *P* = 0.005, t = 2.898). T-Value graphs from Fp2 and Cp4 sensors located in the right fronto-polar (**b**) and right central-parietal regions (**c**). In the individual graphs, significant differences were found in the alpha^2^ and beta^3^ frequencies typically between 10 and 25 Hz where t-Values exceeded the threshold of 2.9

For the EO Group Averaged Differences (*N* = 18), the data were generated by subtracting the POST condition averaged EEG from the respective averaged BL condition EEG samples. From these differences, we compared the location and peak frequency distributions using topographic mapping to display the patterns of change across the three test periods. The paired student’s *t*-Test was then applied to the Group Averages to assess the probability that the differences between the means were not due to chance. The *t*-Test computations were generated as Group Averaged t-Value datasets Fig. 3, where the graphs plot the t-Values of the frequency-by-amplitude differences in the EC EEG from all subjects combined, where t-Values of 2.898 or greater were significant (*N* = 18, df. = 17, *p* = 0.005). The enlarged graphs in Fig. 3 were organized with t-Values on the y-axis and EEG frequencies along the x-axis. In each graph, the t-Values represent the significance of the change measured from the BL to POST period. For the data from Fp2 (right fronto-polar cortex) and Cp4 (right central-parietal cortex), the t-Values from the before vs. after intervention differences were significant in both the alpha and beta bands between 12 and 25 Hz, while several other sites in the right hemisphere indicated significant gamma band differences out to 40 Hz.

**Fig. 4:**
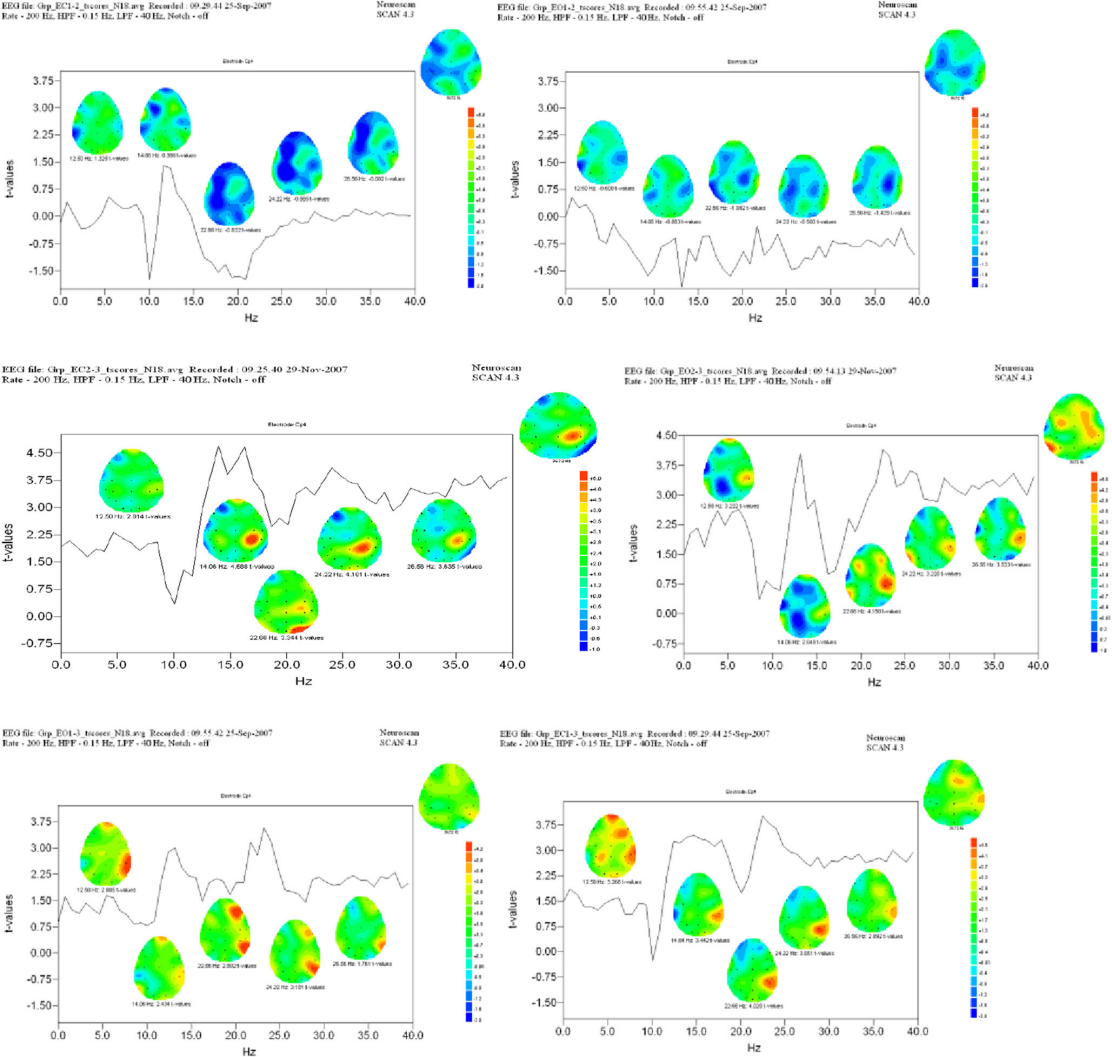
*T*-test results of 1st, 2nd half, and overall comparisons. Graphs of the *t*-Test scores of the differences at the Cp4 site under EC (*left*) and EO (*right*) conditions, where plots show significant changes at 12.5, 14.8, 22.7, 26.6, and 36.7 Hz. The top figures are from the first-half indicating no significant differences from the baseline to middle test periods. The middle figures plot the second-half difference scores from the middle to post test periods, indicating significant reductions in activity (between 12 and 40 Hz) in the fronto-polar, pre-frontal, temporal, parietal, and occipital cortices (*Yellow* to *Red*). The bottom figures display statistical results of the overall differences between the baseline and post periods, where significant changes are predominantly located in the right hemisphere in fronto-polar, prefrontal, and superior- and temporal-parietal cortices in both EC and EO conditions

Figure 4 displays the *t*-Test results of the first-half, second-half, and overall comparisons, where the topographic maps paint the t-Values by location and frequency at 12.5, 14.8, 22.7, 26.6, and 36.7 Hz. Here, values above a threshold of 2.9 (Yellow to Red) indicate a significant result (*P* = 0.005). T-Values from the first-half difference tests (∆^1^) are displayed on the top row (EC – Left Column; EO – Right Column) where no significant changes occurred during that period. The middle graphs (∆^2^) display the second-half results, where significant power increases were evident from the MID sample period compared to the POST. Another way of saying this is that the peak changes were in locations, and networks, that produced less energy after the CBT intervention than before. The ∆^2^ dataset provided clear evidence of the differential involvement of the right orbital-frontal, right vlPFC including the insula, as well as in the right superior-temporal sulcus involving the parietal and temporal cortices at Fp2, F4, F8, P4, and T6 sites. The lower t-Value graphs (∆^3^) show a nearly identical spatial-frequency pattern overall as in the second-half, however significant reductions found in the POST period data were visible as well in midline and contralateral left hemisphere sites at F3, F7, P3 and T5; consistent with the involvement of asymmetric left-hemisphere frontal centers involved in the dialogue process.

**Fig. 5:**
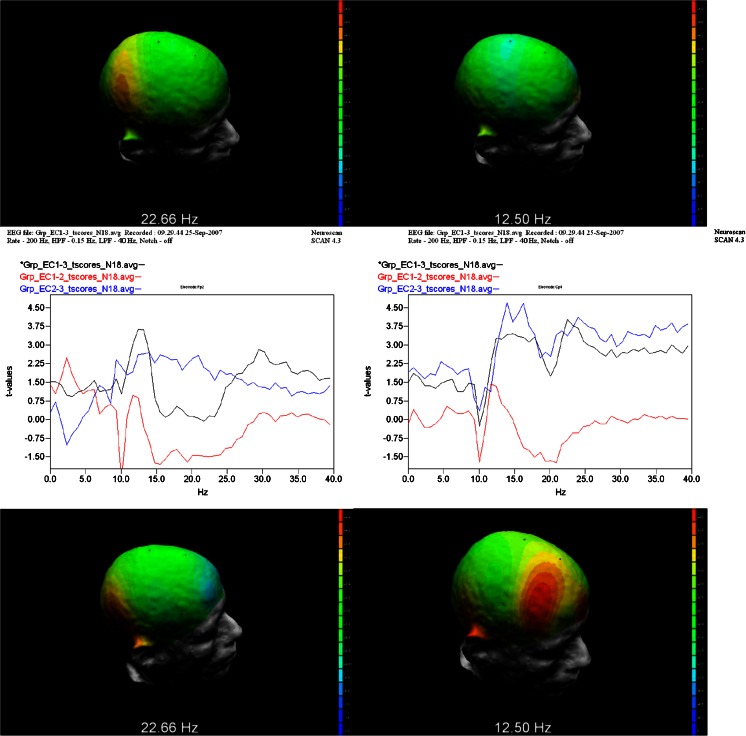
t-Values from 0 to 40 HZ at Fp2 and Cp4 locations. (*Top*) graphs of EC Group *t*-Test results from ∆^1^ (*Black*), ∆^2^ (*Blue*), and ∆^3^ (*Red*) differences for Fp2 (*left*) and Cp4 (*right*) locations. (*Middle*) 3D display of t-Values at Fp2 site at 12.50 Hz (2.61; n.s.) and Cp4 site at 22.66 Hz (3.34) in the ∆^2^ condition. (*Bottom*) display of significant t-Values for Fp2 (3.63) and CP4 sites (4.0) in the ∆^3^ condition

In Figs. 5 (EC) and 6 (EO), the graphs (Top) show the t-Values from 0 to 40 Hz at Fp2 and Cp4 locations for ∆^1^, ∆^2^ and ∆^3^ simultaneously. The t-Values at 12.5 Hz (alpha_2_) and 22.7 Hz (beta_3_) for the ∆^2^ and ∆^3^ test are graphically displayed topographically on a realistic head model. These results provide a 3D view by mapping the t-Values of the differences over the outline of the head, showing a graphical comparison of the peak frequency at each sensor location. Locations colored Yellow to Red indicate significant peak frequencies above 2.9 t-Values. Hence, under both the EC and EO conditions at t-Values of 2.9 or greater, there were significant reductions in power at key brain regions consistent with the locations of the CE, DM and SN networks; and these networks were differentially activated after participation in the 90-day CBT intervention as compared to the BL and MID test periods.

**Fig. 6:**
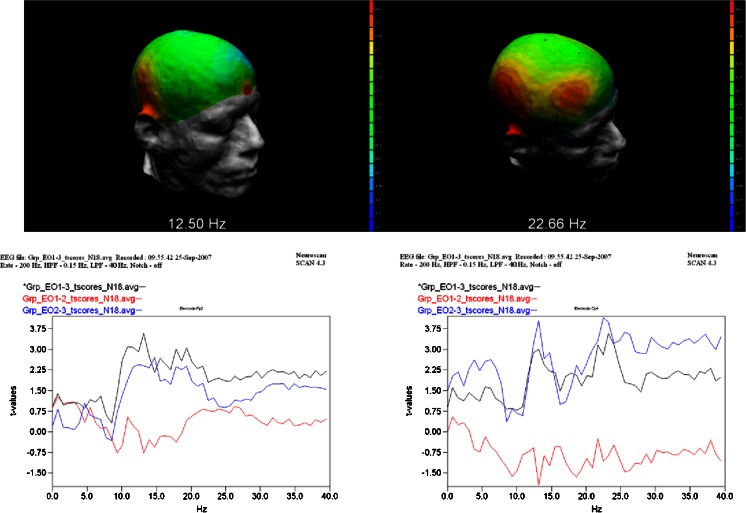
EO Group *t*-Test results from BL mid and post with 3D display of t values at (*Top*) graphs of EO Group *t*-Test results from ∆^1^ (*Black*), ∆^2^ (*Blue*), and ∆^3^ (*Red*) differences for Fp2 (*left*) and Cp4 (*right*) locations. (*Middle*) 3D display of t-Values at Fp2 site at 12.50 Hz (2.44; n.s.) and Cp4 site at 22.66 Hz (4.15) in the ∆^2^ condition. (*Bottom*) display of significant t-Values for Fp2 (2.91) and CP4 sites (4.56) in the ∆^3^ condition

## Discussion

The asymmetric left brain function includes processes involved in the identification of self, whereas the right brain is implicit in the association of one’s-self with one’s surroundings, and with others. In approximately 90 % of right-handed and 70 % of left hand dominant people, the left hemisphere is instrumental in the understanding and use of language (Drane et al. 2012). Mood, imagery and imagination, and functions related to mathematics tend to be co-located within the right hemisphere. However, without complex interactions of the receptive and expressive language systems with the right-brain perceptual and emotional systems, verbal communication using dialogue would be impossible. The right brain, particularly the frontal lobes, is responsible for prosody (i.e., how we see and hear another person in terms of emotional awareness) and empathy. Without prosody, there can be no empathy as empathy implies an emotional bonding or a feeling of something another is experiencing (Cox et al. 2012). Today’s human relationships are constructed on this cross-systems premise, which is a key component of our study using the IRT dialogue based intervention and the basis for our investigation into the network dynamics involved in learning dialogue skills and changing a sense of connection to one’s partner.

Although weaknesses in the study design do exist, we call out that it is a preliminary investigation that is prospective in nature and not intended to be authoritative on the subject of EEG neuroimaging for behavioral investigations. Thus, for a comparison with other larger studies, our investigation lacked an age-matched control group, which made it difficult to control for population errors. Additionally, the individual data were not evaluated with respect to handedness, language dominance, or gender differences, which may have impacted our group averaged results. Additionally, the outcomes were not presented as to whether the individual relationships of the couples within the group lead to an improved situation or ultimate divorce. Lastly, no methods for statistical corrections for multiple within subject measures used in the preparation of the raw EEG data were reported.

Although, such weaknesses must be addressed in future studies and publications, given the early nature of this research effort, we feel it sufficiently important to report these consistent spatial-frequency EEG results from a therapeutic intervention as there is benefit to a broader community of researches investigating brain changes from a host of CBT methodologies.

For this effort, we assumed the 90-day CBT Imago intervention targeted specific brain networks involved in learning and using a new dialogue process; and in particular, that there should be a significant effect in right-brain systems involved in prosody and dyadic adjustment with one’s partner as well as in left brain systems involved in language processing. This report describes the spatial-frequency QEEG analyses used to investigate fine grained RSN changes before, during, and after the CBT intervention and found significant differences at several key spatial-frequency nodes that share connections in the DM, CE and SN networks. We have demonstrated that stable cross-frequency patterns were present in the EEG and theorize such coupling mechanisms to be the means by which the brain makes persistent circuit reorganization; ultimately leading to lasting behavioral changes. These results show a distributed network pattern of activity in the alpha_2_ band coupled at beta_3_ and gamma frequencies with nodes located in the fronto-polar, pre-frontal, and superior temporal-parietal integrative cortices; which also include connections into the right hippocampus and cuneus that influence attention, emotional memory, and regulation of mood. We also identified significant left hemisphere alpha_2_ and beta_3_ differences in sites overlying both Wernicke’s and Broca’s areas that we expect are involved in learning and using the verbal dialogue method.

Interestingly, no significant spatial-frequency differences were found over the first-half of the program in any brain systems, which is likely due to the time required for learning the dialogue process and developing a strategy for achieving the course goals of an improved relationship dynamic. All significant differences in location and frequency occurred during the second-half of the CBT program, which were also identified in the overall assessment. Thus, with an understanding of: 1) *where to look* (e.g., left-frontal and temporal-parietal cortex for language; the right pre-frontal, insular, and superior temporal-parietal cortex for prosody and empathy and the frontal-polar and superior-parietal connectivity for executive function, decision-making and mood regulation), and 2) *what frequencies to look at* (e.g., in the high alpha and beta frequencies, coupled with persistent gamma band activity), we successfully identified consistent differences in spatial-frequency EEG patterns over three independent test periods that were evaluated using the Student’s *t*-Test for independent means. Graphs and topographic maps were then used to display the locations and peak frequencies of the power differences as well as the independent *t*-Test results.

DAS scores provide limited information about changes due to the low number of subjects, which led to a limited statistical strength of the determination of a significant difference. The analysis of individual couple’s scores showed that some had large changes in dyadic adjustment coincident with an overall reduction in distress over the intervention. Six couples increased DAS scores while two couples received lower scores, and one couple stayed the same. The loss of points might be related to recognition that relationship improvement was not to be realized for these two couples in the course of this program. We offer the information about the DAS not as a measure of correlation to the brain changes, but as secondary source of couple information to suggest what might be happening with the areas we observed in the system level spatial-frequency EEG changes.

Fung and Morris (2000) state that “it emerges that a word and its associated concepts, rather than being encoded as one discrete anatomical unit, are coded in multiple regions of the brain”. Thus, we conclude that language processing is therefore distributed rather than strictly localized, and in hearing a word or phrase in conversation, what likely ensues is activation of a large number of distinct and interconnected neurons, distributed widely in the brain, which together allow its correct interpretation and use.

In keeping with the idea of distributed functions in the hearing and language areas, research has found no structural, anatomical, or physiological criteria that precisely defined the boundaries of key brain nodes (e.g., Heschl’s, Broca’s and Wernicke’s areas) (Bauer et al. 2003). With the exception of word deafness and aphemia, correlation between language deficits and the anatomical location of lesions is quite imprecise. Neither Broca’s nor Wernicke’s aphasias are pure deficits of comprehension and language output, which suggests that interpretation of meaning, word selection and organization into correct syntax are not sequential processes taking place uniquely in either node.

Furthermore, a number of structures outside these two areas, including the supplementary motor area and basal ganglia, are implicated in the processing of speech output, as evidenced from lesion studies (Thatcher et al. 2009). Therefore language comprehension and production is probably not a serial process, but instead involves parallel activation of multiple pathways using large scale neurocognitive networks spanning multiple interconnected nodes with multiple iterative or reentrant circuits. The clinical, neuroanatomical, and neurophysiological evidence is expansive and broadly in agreement with these beliefs (Guerra-Carillo et al. 2014; Lang et al. 2014; Ganzetti and Mantini 2013).

Additionally, both neuropsychological and neuroimaging studies show that cortical and subcortical networks are involved in attention, motivation, executive function, pleasure-seeking behavior, and emotional regulation. Some researchers have even provided schematics of the oscillators within the brain along with detailed descriptions of how a system-of-nodes manages homeostatic regulation, sensory-motor integration and higher order cognitive function (Lau et al. 2013; Aziz-Zadeh et al. 2010).

Further research has shown that multiple brain processing nodes are involved in maintaining emotional regulation and changing decision-making strategy that are the same as those identified here (Vogels and Abbott 2009). In another study by Cohen et al., areas of the brain related to reversals of negative behaviors activated both sides of PFC in an area near the insula to successfully bring about a behavioral change (Cohen et al. 2009). When reviewing the emotional brain literature, it is interesting to see the overlapping relationships among the DM, CE, SN, and as well, the language and reward systems. In the DM and reward systems for instance, the former is separate from the latter because the ventral tegmentum, nucleus accumbens (NA), and striatum are not included in the DM network (Thatcher et al. 2009). However, the NA sends fibers to the septal nuclei in the orbital-frontal cortex that sends fibers to the posterior hippocampus, which is part of the DM. And, at the same time, the DM sends fibers to the anterior hippocampus, through numerous connections in the cingulate cortex that also project to the hypothalamus - thus forming the Papez circuit. This system works as a double-feedback loop of systems that share a common node (i.e., the hippocampus or cingulate). Therefore, we argue that such network patterns of activity are well suited in nature and persistent changes in the spatial-frequency activity in these networks likely lead to persistent changes in behavior.

## Conclusion

Discovering more information about the internal mechanisms of the brain involved with participation with interventions designed to help distressed couples is useful in illuminating ways that may be helpful to alleviate inherent social and physical problems connected to relationship distress. This study joins other efforts beginning to explore the greater possibilities for utilizing QEEG as a low cost tool for assessing treatment outcomes in educational and counseling settings. This study offers preliminary information about system level spatial-frequency EEG changes in couples who participated in a 90-day CBT program for couples, as well as attending a group counseling program designed to support the dialogue based activities learned in the couple’s workshop.

With a primarily Caucasian, high income group of nine couples, all but two of the couples started out in a distressed category and all but three couples improved scores in dyadic adjustment coincident with progressive changes in a targeted reduction of power in specific brain locations after the intervention. Overall, the types of DAS changes reported were found to be consistent with behavioral actions attributed to the same brain networks observed to show reduced power in the system level spatial-frequency EEGs.

It is important to note that none of the couples participated in any other treatment besides the Imago program and did not take psychotropic medications or illegal drugs during the study time frame. Thus, we have demonstrated in a small scale prospective experiment that low-cost QEEG neuroimaging methods are available today with a high degree of temporal and spatial resolution sufficient to identify statistically significant differential brain activity after a 90-day couple’s relationship improvement program. Primarily important is that the brain locations and frequencies identified as coincident with learning Imago dialogue skills and using an empathy-based process are consistent with our understanding of cooperative brain systems and how they coordinate to perform complex cognitive tasks and ultimately change behaviors. Therefore, we believe that continued research is warranted to continue the preliminary investigation of brain changes in CBT and further examine the potential of spatial-frequency quantitative EEG approaches for imaging fine-grain neural activity during complex neurocognitive paradigms.
